# Expression of nuclear receptor co‑activator 7 protein is associated with poor prognosis of breast cancer

**DOI:** 10.3892/ol.2024.14411

**Published:** 2024-04-23

**Authors:** Ziluo Peng, Yanlin Li, Song Xia, Qian Dai, Liang Yin, Miao Chen, Wannian Yang, Genbao Shao, Qiong Lin

**Affiliations:** 1 School of Medicine, Jiangsu University, Zhenjiang, Jiangsu 212013, P.R. China; 2 Department of Clinical Laboratory, The First Affiliated Hospital of Anhui Medical University, Hefei, Anhui 230022, P.R. China; 3 Department of Breast Surgery, Jiangsu University Affiliated People's Hospital, Zhenjiang, Jiangsu 212050, P.R. China; 4 Department of Pathology, Jiangsu University Affiliated People's Hospital, Zhenjiang, Jiangsu 212050, P.R. China

**Keywords:** NCOA7, ERAP140, breast cancer, biomarker, poor prognosis, metastasis

## Abstract

Nuclear receptor coactivator 7 (NCOA7) is an estrogen receptor binding protein. Its role in breast cancer progression has so far remained elusive. The present study aimed to determine the expression levels of NCOA7 in breast tumor samples and confirmed its potential utility as a breast cancer prognostic biomarker. The expression of NCOA7 was detected by immunohistochemical staining in 241 breast cancer tumor samples and 163 adjacent normal tissue samples. The association of NCOA7 expression with the clinicopathological characteristics and overall survival were statistically analyzed. Cell proliferation was determined by Cell Counting Kit-8 and colony-formation assays. Cell migration was detected using wound-healing and Transwell assays. NCOA7 was positively expressed in 44% of breast tumor tissues. The expression of NCOA7 was positively associated with tumor size (T-stage; P=0.005) and lymph node metastasis (N-stage; P=0.008). Additional statistical analysis indicated that the expression of NCOA7 was associated with patient age, tumor size and lymph node metastasis in patients with triple-negative breast cancer (TNBC) compared with that in patients with non-TNBC. The overall survival of patients with NCOA7-positive breast cancer was significantly lower than that of patients with NCOA7-negative breast cancer (P*=*0.006). Among the patients with lymph node metastasis, the overall survival was reversely associated with the expression of NCOA7 (P=0.042). Furthermore, knockdown of NCOA7 expression in breast cancer T47D and MCF7 cells significantly inhibited both cell proliferation and migration, suggesting that this protein may exert a role in driving breast cancer progression. Taken together, these results indicate that the expression of NCOA7 is associated with poor prognosis of breast cancer and suggest that this protein may be a driver for metastasis and a potential therapeutic target for advanced breast cancer.

## Introduction

Breast cancer is a common malignant disease in the female population with high numbers of mortality (1–3). Although significant progress has been made in breast cancer research and anti-hormone treatment in recent years, the survival rate of patients with advanced-stage disease remains poor (4,5). The identification of new biomarkers of prognosis and therapeutic target molecules and the development of effective therapeutic drugs for advanced breast cancer are necessary for improving the survival rate of patients with this disease, particularly with advanced breast cancer.

Nuclear receptor co-activator 7 (NCOA7, also named ERAP140) is a member of the NCOA family and binds to the estrogen receptor (ER) (6). NCOA7 also belongs to the oxidation-resistant 1 (OXR1) protein family that contains the Tre2/Bub2/Cdc16, lysin motif, domain catalytic (TLDc) domain and may serve as an anti-oxidation protein against oxidative stress (7,8). Previous studies have shown that NCOA7 interacts with [H^+^]-V-ATPase through its TLDc domain and may regulate endosomal vesicle trafficking and viral entry (9–12). In addition, NCOA7 inhibited the MAPK/ERK pathway to regulate epithelial-mesenchymal transition and apoptosis, thereby inhibiting the progression of clear-cell renal cell carcinoma (13); it is a potential biomarker in oral squamous cell carcinoma and a member of prognostic indicators for neuroblastoma and colon adenocarcinoma (14–16). Genetic variations of the *NCOA7* gene are associated with a reduced risk of breast cancer (17). However, the role of NCOA7, as an ER-binding protein, in regulating breast cancer progression has remained elusive. To the best of our knowledge, the expression of NCOA7 in breast tumor tissues and its association with clinicopathological characteristics and the survival rate of patients with breast cancer have remained to be investigated.

To fully understand the role of NCOA7 in breast cancer progression, the current study set out to determine the expression levels of NCOA7 in tumors from patients with breast cancer and the association of NCOA7 expression with the survival of these patients. The results indicated that NCOA7 was overexpressed in breast tumors and that its expression was reversely associated with the survival of patients with breast cancer, suggesting that it may be a biomarker for poor prognosis of breast cancer.

## Patients and methods

### Materials and patient samples

The following materials were used: Anti-NCOA7 antibody (cat. no. 393427; 1:300 dilution for immunohistochemistry and 1:500 dilution for western blot analysis; Santa Cruz Biotechnology, Inc.), anti-β-actin antibody (cat. no. 100166-MM10; 1:500 dilution; Zoonbio Biotechnology), immunohistochemical (IHC) reagents including formalin, hydrogen peroxide, xylene, ethanol, trisodium citrate and citric acid (Shanghai Sinopharm Chemical Co.), Cell Counting Kit-8 (CCK-8; Vazyme Biotech Co. Ltd.), crystal violet (Beyotime Institute of Biotechnology), diaminobenzidine (DAB) color development (ZSGB-BIO; OriGene Technologies, Inc.), the human breast cancer cell lines T47D (cat. no. HTB-133) and MCF7 (cat. no. HTB-22) and the human embryo kidney cell line 293T (cat. no. CRL-3216; American Type Culture Collection). The breast cancer tissues used in the present study were selected from a breast cancer sample deposit library from patients with newly diagnosed breast cancer who visited the Breast Surgery Department of Jiangsu University Affiliated People's Hospital (Zhenjiang, China) between December 2010 and October 2016. The paraffin-embedded breast cancer tissue microarray (TMA) chips containing 241 tumor tissue samples and 163 adjacent normal tissue samples and related clinicopathological information of the patients used in the present study were obtained from the Department of Pathology of Jiangsu University Affiliated People's Hospital (Zhenjiang, China). All tissue specimens collected were from patients who provided written informed consent. The study protocol was approved by the Institutional Review Board of Jiangsu University Affiliated People's Hospital (Zhenjiang, China; approval no. LLYW20210004). All breast cancer tissue donors were female and did not receive any treatment prior to the surgery. The follow-up for patient survival information was performed by telephone. Information regarding patient age, tumor size, lymph node metastasis (N-stage), and tumor, node and metastasis (TNM) stages is presented in Tables I and SI. The median age of all patients was 53.0 years. Furthermore, the median age was 53.0 years in patients with triple negative breast cancer (TNBC) and 53.5 years in patients with non-TNBC (N-TNBC). There was no significant age difference (P=0.938) between the TNBC and the N-TNBC groups. Overall survival was defined as the time from surgery to patient death from any cause.

### IHC staining of the TMA

The paraffin-embedded breast cancer or normal tissue microarray chips were baked at 65°C for 1 to 2 h. The specimens were subsequently dewaxed by immersion in xylene for 30 min, followed by hydration using gradients of alcohol (concentrations of 100, 95, 80, 70, 50%) for 5 min each at room temperature. The specimens were completely immersed in 800 ml sodium citrate buffer (pH 6.0) for antigen repair, boiled in a microwave oven for 20 min and then cooled naturally to room temperature. Incubation with 3% hydrogen peroxide at room temperature for 10 min was performed to block endogenous peroxidase activity and non-specific protein interactions. The samples were subsequently incubated with NCOA7 antibody (rabbit; cat. no. 393427; 1:300 dilution; Santa Cruz Biotechnology, Inc.) overnight at 4°C and then with biotinylated goat anti-rabbit secondary antibody working solution (cat. no. SA1020; Boster Biological Technology Co. Ltd.) at 37°C for 30 min, followed by color development with DAB. The slides were visualized under light microscopy and the reaction was terminated immediately when a yellow pellet was present. The staining was repeated with hematoxylin for 30 sec at room temperature. The specimens were subsequently dehydrated in a gradient of alcohol (50, 70, 80, 95 and 100%) for 5 min each, followed by immersion in xylene for 30 min for transparency at room temperature. Finally, the films were sealed with resin glue. Phosphate buffer (5% BSA; Sigma-Aldrich; Merck KGaA) was used instead of primary antibodies to prepare the negative controls and positive tissue sections with specific staining expression were used as positive controls. The IHC staining assay was performed as previously described (18).

### IHC staining scoring

Semi-quantitative scoring was performed by two independent observers. The immunostaining score was determined according to the positivity rate and staining intensity and was scored according to the immuno-reactive score (19), based on the percentage of positive cells: 0 (≤5%), 1 (5–25%), 2 (25–50%), 3 (50–75%) and 4 (≥75%). The staining intensity was scored as follows: 0 (no staining), 1 (yellowish), 2 (yellow) and 3 (brownish). The NCOA7 immunostaining score was then calculated as follows: Percentage positive score × staining intensity score. A score ≥4 was considered to indicate positive expression.

### Cell culture

T47D and MCF7 cells were cultured in RPMI-1640 (cat. no. 350-006-CL) and 293T cells were cultured in DMEM [cat. no. 319-005-CL; both from Wisent Biotechnology (Nanjing) Co. Ltd.] supplemented with 10% FBS (cat. no. 40130ES76; Yeasen Biotechnology Co., Ltd.), 100 U/ml penicillin and 100 mg/ml streptomycin at 37°C in the presence of 5% CO_2_ and 90–95% relative humidity.

### Knockdown of NCOA7 expression in breast cancer cells by lentiviral vector-loaded short hairpin RNAs (shRNAs)

shRNA of luciferase (shLuc; target sequence: 5′-CGCTGAGTACTTCGAAATGTC-3′) was used as a control. The nucleotide oligos of two NCOA7 shRNAs (Sangon Biotech Co., Ltd.) were synthesized and cloned into the lentiviral shRNA expression vector TETO-pLKO.1-TRC (Addgene, Inc.), in which expression of shRNA was induced by doxycycline. The shRNA oligos were inserted into the *Age*I/*EcoR*I sites of the vector (20).

Two NCOA7 shRNA sequences were used as follows: shNCOA7-1 forward, 5′-CCGGTTGCGCTCTACAATGACATTTCTCGAGAAATGTCATTGTAGAGCGCAATTTTTG-3′ and reverse, 5′-AATTCAAAAATTGCGCTCTACAATGACATTCTCGAGAAATGTCATTGTAGAGCGCAA-3′; shNCOA7-2 forward, 5′-CCGGCCTGTGAGAAGCAAGATATAACTCGAGTTATATCTTGCTTCTCACAGGTTTTTG-3′ and reverse, 5′-AATTCAAAAACCTGTGAGAAGCAAGATATAACTCGAGTTATATCTTGCTTCTCACAGG-3′.

The lentiviral shRNA plasmid was co-transfected with psPAX2 (cat. no. 12260; Addgene, Inc.) and pMD2.G (cat. no. 12259; Addgene, Inc.) in 293T cells for 8–10 h. The transfection medium was then replaced with 2 ml culture medium. Following 24 h, the culture medium containing viral particles was collected every day for three days. The collected medium was cleared by centrifugation at 1,250 × g for 5 min at 4°C and stored at 4°C for use. To transfect T47D and MCF7 cells, 6 µg/ml polybrene was added along with the viral particle-containing medium (multiplicity of infection, 12) and selected with puromycin. The NCOA7 knockdown effect was determined by western blot analysis with an anti-NCOA7 antibody (1:500 dilution). Lentiviral construction and infection were performed as previously described (21).

### Preparation of breast cancer cell lysates, gel electrophoresis and western blot analysis

The cells were washed with precooled PBS, lysed by adding an appropriate amount of mammalian cell lysis buffer [40 mM Hepes (pH 7.4), 100 mM NaCl, 1% Triton X-100, 25 mM glycerol phosphate, 1 mM EDTA, 1 mM sodium orthovanadate, 10 µg/ml leupeptin and 10 µg/ml aprotinin] and shaken slowly at 4°C for 30 min. The cell lysates were transferred to an Eppendorf (EP) tube and centrifuged for 15 min at 12,000 g at 4°C. The cleared lysates were transferred to a clean EP tube, mixed with 5X SDS buffer and boiled at 100°C for 8 min. The proteins of the lysates were separated by 10% SDS-PAGE and subsequently transferred to a polyvinylidene fluoride membrane (cat. no. IPFL00010; EMD Millipore). The transferred membrane was blocked with 1% BSA for 1 h at room temperature and incubated with primary antibodies (NCOA7 or β-actin; 1:500 dilution) at 4°C overnight. Following washing with 1X Tris-buffered saline containing Tween-20, the membrane was incubated with horseradish peroxidase-conjugated secondary antibody (anti-mouse; cat. no. 31430; 1:10,000 dilution; Thermo Fisher Scientific, Inc.) at room temperature for 1–2 h. The protein bands on the membrane were detected by an electrochemiluminescence detection kit (cat. no. P0018M; Beyotime Institute of Biotechnology). Western blot analysis was performed as previously described (22).

### Cell proliferation assay

Cell proliferation was determined by the CCK-8 assay. The control or NCOA7-knockdown T47D cells (1×10^4^/well) or MCF7 cells (8×10^3^/well) were seeded in a 96-well plate. Following cell culture for the indicated durations, the CCK-8 reagent was added. The cells were cultured for 2 h and the cell density was detected by measuring the absorbance at 450 nm using a microplate reader.

### Colony-formation assay

The control or NCOA7-knockdown T47D (1×10^3^/well) or MCF7 cells (0.5×10^3^/well) were seeded in a 6-well culture plate. Following culture for 14 days, the cell colonies were fixed with 4% paraformaldehyde and stained with 0.1% crystal violet solution for 8 min at room temperature. Following washing three times with PBS, the stained cell colonies were counted.

### Wound-healing assay

Cell migration was determined by the wound-healing assay. T47D cells (6×10^5^/well) or MCF7 cells (5×10^5^/well) were seeded in a 12-well plate. Following culture for 12 h, a straight scratch line was performed at the center area of the cell monolayer using a pipette tip. The cell layers were then rinsed to remove any detached cells and debris. The cells were then cultured with fresh DMEM. Images of the scratch line were captured following cell culture for 24 or 48 h after the scratch line was created under a phase-contrast microscope. The migrated area of the cells was calculated from the images using the imaging software Image J (version 1.53e; National Institutes of Health).

### Transwell assay

Transwell chambers (cat. no. 3422; Corning, Inc.) were used for migration assays. The cells (2×10^5^ cells/ml) in 200 µl serum-free DMEM were seeded in each top well of the chamber. DMEM with a migration attractant (10% FBS) was added to the bottom well of the chamber. Following incubation under normal culture conditions for 24 h, the cells on the top side of the membrane between the top and the bottom chambers were carefully removed and the cells that had migrated to the bottom side of the membrane were fixed with 4% paraformaldehyde for 30 min and stained with 0.1% crystal violet solution for 8 min at room temperature. The stained cells were washed with PBS three times, visualized under a phase-contrast microscope and counted. Cell proliferation and migration assays were performed as previously described (20).

### Statistical analysis

SPSS 22.0 statistical software (IBM Corp.) was used for statistical analysis of the data. The association of NCOA7 expression with the survival of patients with breast cancer was statistically analyzed by Kaplan-Meier (K-M) survival analysis with the log-rank test. An independent-samples t-test was used to assess differences between two groups. Differences between multiple groups were analyzed using one-way ANOVA followed by Tukey's honestly significant difference post-hoc test. Data are expressed as the mean ± standard error of the mean. The χ^2^ test was used to analyze the clinicopathological data of the patients in Table I, Table II, Table III and SII. P≤0.05 was considered to indicate a statistically significant difference.

## Results

### NCOA7 is overexpressed in breast tumor tissues

**Figure 1.**
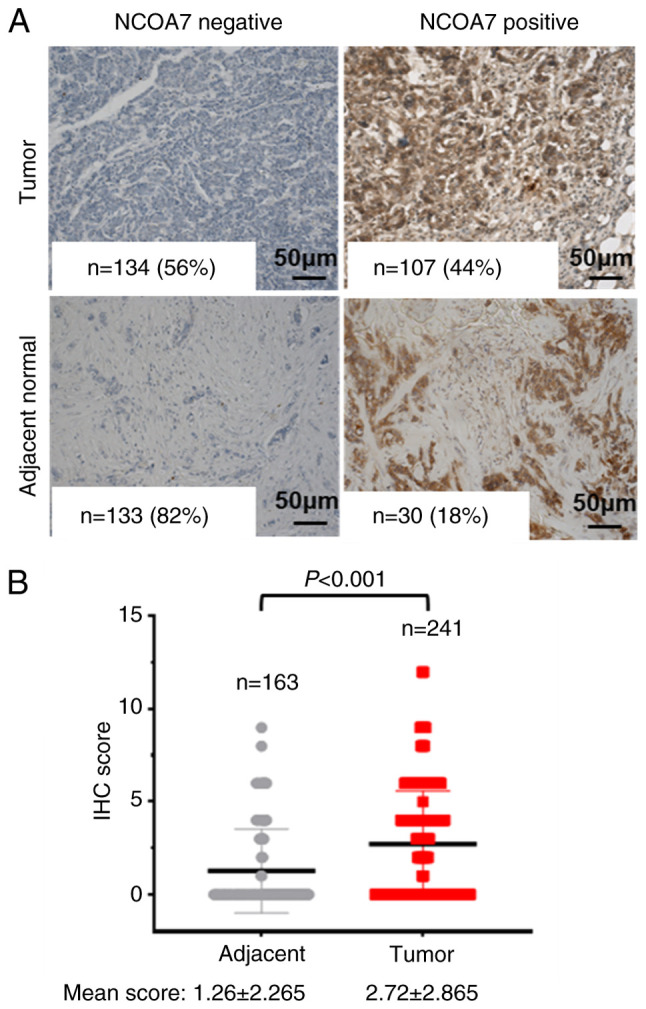
NCOA7 is overexpressed in breast tumors. (A) The expression of NCOA7 was detected by IHC (scale bars, 50 µm). (B) Dot-plot of IHC staining scores of NCOA7 in 241 breast tumor and 163 adjacent normal tissue samples. n indicates case numbers. NCOA7, nuclear receptor coactivator 7; IHC, immunohistochemistry.

To address the role of NCOA7 in breast cancer progression, its expression was examined in a TMA containing 241 breast tumor tissues and 163 normal tissue samples (adjacent to the tumor tissue) by IHC staining with an anti-NCOA7 antibody. The results indicated that NCOA7 was expressed in 107 out of 241 breast cancer tumor samples (44%) and in only 30 out of 163 adjacent normal tissue samples (18%; Fig. 1A). The mean score of the IHC staining in the breast tumor samples was 2.72±2.865, while in the normal tissue samples, it was 1.26±2.265 (Fig. 1B). Statistical analysis of the difference in expression of NCOA7 between the breast tumor and the adjacent normal tissue samples indicated that the expression of NCOA7 in breast tumor tissues was significantly higher than that in the adjacent normal tissues (P<0.001; Fig. 1B).

### Expression of NCOA7 is positively associated with tumor size, N-stage and T-stage of breast cancer

Subsequently, the association of the expression of NCOA7 with the clinicopathological parameters was determined by statistical analysis of the TMA data. As presented in Table I, the expression of NCOA7 was positively associated with the tumor size (P=0.020), T-stage (P=0.005) and N-stage (P=0.008). However, no significant association was observed for the age of the patients (P=0.914) and the TNM stage (P=0.299). Among the samples of TNBC, the expression levels of NCOA7 were significantly associated with the N stage (P=0.032), while they were not associated with any other parameters (Table II). However, the expression levels of NCOA7 in samples of N-TNBC did not exhibit any significant association with any of the clinicopathological parameters examined (Table III).

Furthermore, the expression levels of NCOA7 were compared in each pathological parameter of TNBC and N-TNBC. As shown in Table SII, the expression of NCOA7 was significantly higher in patients with TNBC than in patients with N-TNBC who were aged >50 years (P=0.023), with a tumor size of >3 cm (P=0.007) and those who had lymph node metastasis stages N1-3 (P=0.023). These data suggested that the expression levels of NCOA7 were preferentially associated with the pathological parameters related to advanced breast cancer in TNBC compared with N-TNBC.

### Expression of NCOA7 is inversely associated with overall survival of patients with breast cancer

**Figure 2.**
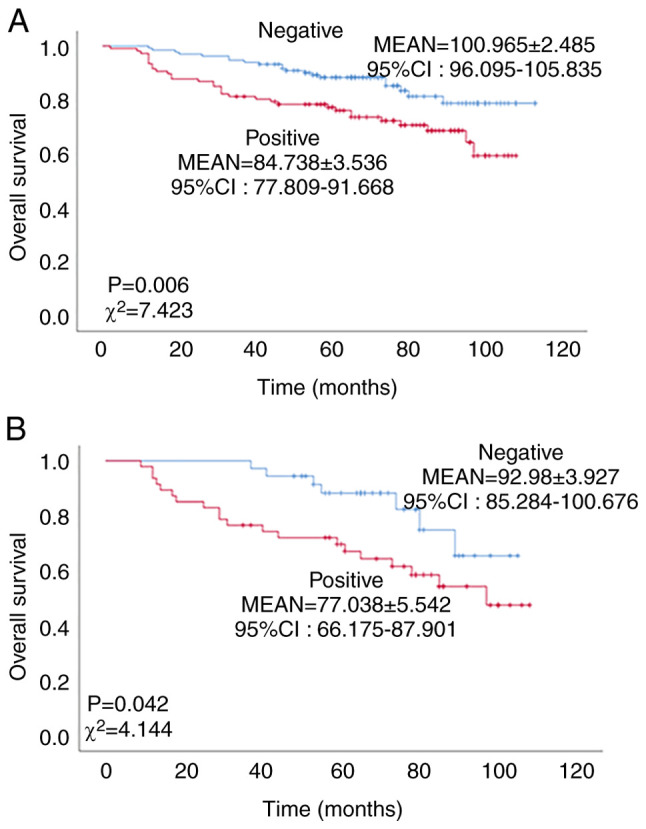
NCOA7 expression is inversely associated with overall survival of patients with breast cancer. (A) K-M survival analysis of the association between expression of NCOA7 and overall survival was performed for all patients with breast cancer. (B) K-M survival analysis of the association between the expression levels of NCOA7 and overall survival was performed for patients with lymph node metastasis. The data used for statistical analysis were derived from the immunohistochemical staining analysis of 86 breast tumor samples derived from the patients with N1-3 lymph node metastasis, including 38 NCOA7-negative and 48 NCOA7-positive samples. The values printed in the figure are the mean overall survival time in months. NCOA7, nuclear receptor coactivator 7; K-M, Kaplan-Meier.

The association between the expression of NCOA7 and the overall survival of patients with breast cancer was determined by K-M analysis. As shown in Fig. 2A, the overall survival of the NCOA7-positive patients with breast cancer was significantly lower than that of the NCOA7-negative patients (χ^2^=7.423, P=0.006). The mean survival time of NCOA7-positive patients with breast cancer was 84.738±3.536 months, while that of the NCOA7-negative patients was 100.965±2.485 months. These data indicate that the expression of NCOA7 is inversely associated with overall survival of patients with breast cancer. To determine the role of NCOA7 in breast cancer metastasis, the association of NCOA7 expression with the overall survival of patients with lymph node metastasis (N1-3; total cases, n=86) was statistically analyzed. As shown in Fig. 2B, the expression of NCOA7 in patients with stage N1-3 was significantly associated with lower overall survival (P=0.042, χ^2^=4.144). The mean survival time of NCOA7-positive N1-3 patients was 77.038±5.542 [95% CI: 66.175–87.901] months, while in NCOA7-negative N1-3 patients, it was 92.980±3.927 [95% CI: 85.284–100.676] months.

### Knockdown of NCOA7 expression inhibits breast cancer-cell proliferation

**Figure 3.**
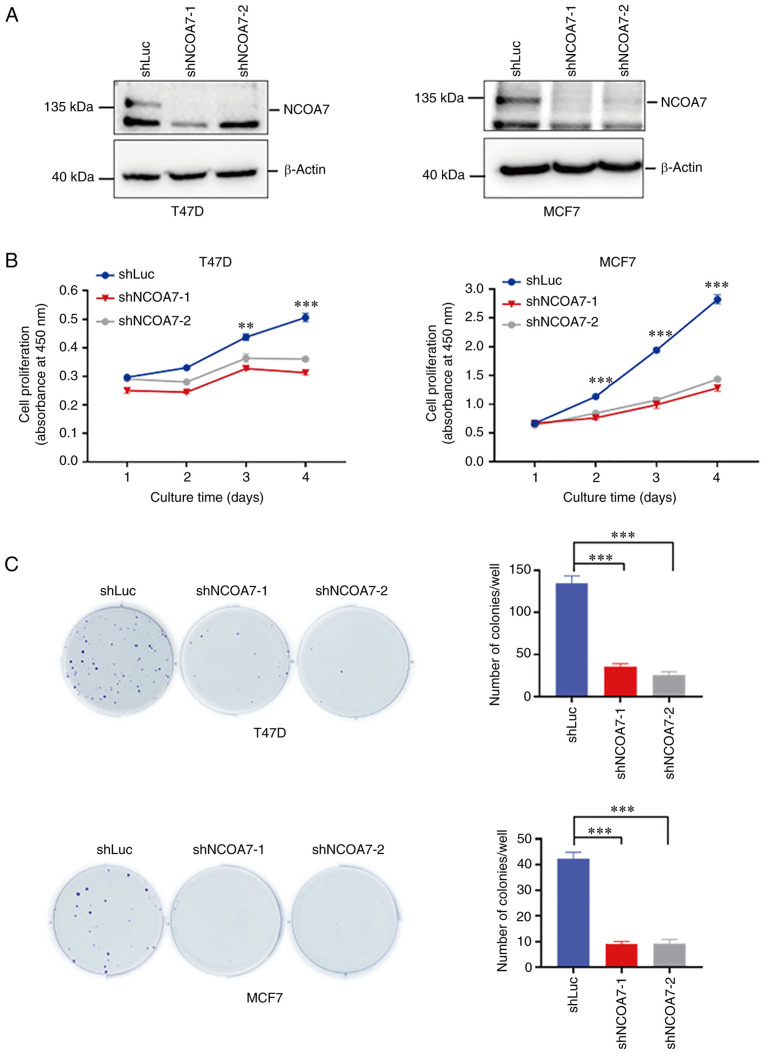
Knockdown of NCOA7 expression in breast cancer T47D and MCF7 cells inhibits proliferation. (A) NCOA7 expression was detected in T47D and MCF7 cells by western blot analysis with an anti-NCOA7 antibody. Β-actin was used as a loading control. (B) The proliferation of T47D and MCF7 cells was detected by the Cell Counting Kit-8 assay within 4 days. The absorbance was detected at 450 nm at the specified times. (C) Images of colony-formation assay using T47D and MCF7 cells. Quantification of the number of colonies per well. **P<0.01, ***P<0.001 vs. shLuc. NCOA7, nuclear receptor coactivator 7; sh, short hairpin RNA; Luc, luciferase (control).

To confirm the role of NCOA7 in promoting breast cancer progression (noted in the breast tumor IHC staining analysis), the effect of knockdown of NCOA7 expression on the proliferation of breast cancer T47D and MCF7 cells was examined. The expression of NCOA7 was knocked down in the breast cancer cell lines T47D and MCF7 by the lentiviral vector loaded with NCOA7 shRNAs along with the control shRNA-transfected cell line that expressed a luciferase shRNA (shLuc) (Fig. 3A). The shNCOA7s were able to deplete ~90% of NCOA7 in both T47D and MCF7 cells (Fig. 3A). CCK-8 and colony-formation assays were performed to determine the effect of knockdown of NCOA7 expression on the proliferation in these cell lines. As shown in Fig. 3B, knockdown of NCOA7 expression by both shNCOA7s consistently inhibited ~80% of cell proliferation following 4 days of culture in both T47D and MCF7 cells, as determined by the CCK-8 assay. However, in the colony-formation assay, knockdown of NCOA7 expression eliminated ~80% of the colony formation in both T47D and MCF7 cells (Fig. 3C), which was consistent with the results obtained by the CCK-8 assay.

### Knockdown of NCOA7 expression severely impairs breast cancer cell migration

**Figure 4.**
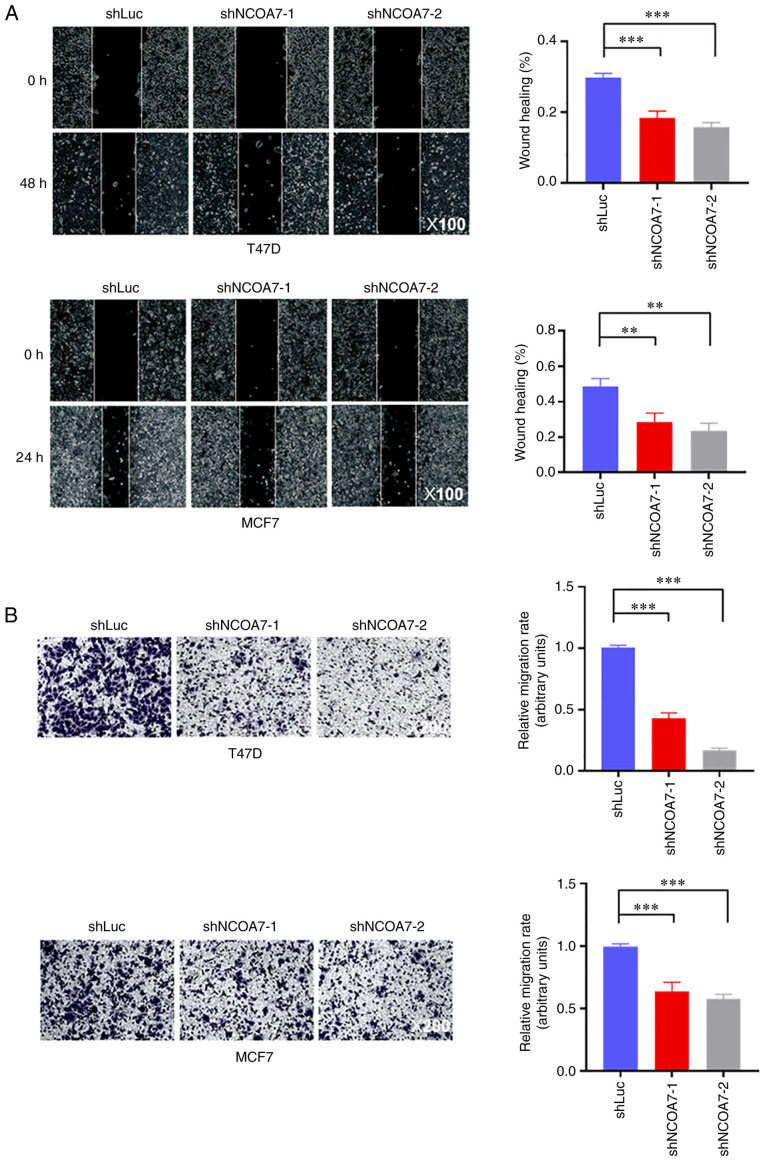
Knockdown of NCOA7 expression in breast cancer T47D and MCF7 cells inhibits their migration. (A) Cell migration was detected by the wound-healing assay. Left panels, representative images of wound healing of confluent control and knockdown cell line layers (magnification, ×100). Right panels, quantification of cell migration by the wound-healing assay with T47D and MCF7 cells. The ratio of the wound-healing area at 24 or 48 h to the scratch area at 0 h was presented. (B) Cell migration was detected by the Transwell assay. Left panels, representative crystal violet staining images of migrated cells on the bottom side of the membrane in the Transwell assays (magnification, ×200). Right panels, quantification of cell migration by the Transwell assay with T47D and MCF7 cells. **P<0.01, ***P<0.001. NCOA7, nuclear receptor coactivator 7; sh, short hairpin RNA; Luc, luciferase (control).

To examine the role of NCOA7 in promoting breast cancer metastasis, the effect of NCOA7 on breast cancer cell migration was determined using the wound-healing and Transwell assays. As shown in Fig. 4A, knockdown of NCOA7 expression by both shNCOA7s reduced the migration rate of T47D and MCF7 cells by ~50%, as determined by the wound-healing assay. Consistent with this, the Transwell assay indicated that knockdown of NCOA7 expression by both shNCOA7s inhibited ~40-50% of cell migration in MCF7 cells, while this percentage was somewhat higher in T47D cells (~60-80%; Fig. 4B).

## Discussion

Establishing prognostic biomarker molecules for patients with postoperative breast cancer is important for the treatment of patients with breast cancer, notably for those with advanced stages of the disease (23,24). In the present study, it was shown that the nuclear receptor (particularly ER) co-activator NCOA7 was overexpressed in breast tumors and its expression was reversely associated with the overall survival of patients with breast cancer. Studies using IHC staining of NCOA7 in breast tumor tissue samples indicated that its expression was associated with tumor size and lymph node metastasis of breast cancer. The data from the breast cancer cell studies (knockdown of NCOA7 expression) indicated that diminishing NCOA7 expression in breast cancer cells inhibited cell proliferation and migration. This confirmed the results obtained from the breast tumor IHC staining studies indicating that the expression levels of NCOA7 were associated with breast tumor growth and metastasis. Therefore, it is proposed that NCOA7 is a prognostic biomarker associated with poor survival of patients with breast cancer. Furthermore, NCOA7 may be considered to be a driver protein for breast tumor growth and metastasis and a potential therapeutic target for breast cancer, notably for advanced breast cancer.

Currently, the molecular mechanism by which NCOA7 promotes breast cancer progression remains to be elucidated. Previous studies have shown that NCOA7 interacts with ERα and potentially functions as an ERα co-activator (17). It was initially expected that NCOA7 may promote breast cancer progression via ER signaling. However, the results of the present study indicate that the expression levels of NCOA7 are preferentially associated with the clinicopathological parameters of TNBC, suggesting that the oncogenic effect of NCOA7 on breast cancer, at least in TNBC, may not be mediated via ER signaling. Notably, in addition to a potential co-activator of ER, NCOA7 may function as an anti-oxidation and vesicle trafficking protein. NCOA7 contains the TLDc domain that is the signature domain of members of the anti-oxidation OXR1 family of proteins (7). Therefore, NCOA7 may exert an anti-oxidative function and protect breast cancer cells from the damage of oxidative stress. However, limited experimental data have shown the role of NCOA7 in oxidation resistance or have examined the TLDc domain required for NCOA7 to promote breast cancer cell proliferation or migration. Recent studies discovered that the TLDc domain of NCOA7 interacted with [H^+^]-V-ATPase and regulated vesicle acidification (9,10). NCOA7 may utilize its function in vesicle trafficking to regulate intracellular oncogenic signaling, particularly endocytotic receptor signaling or secretory signaling. Given these known molecular interactions of NCOA7, it is speculated that it promotes breast cancer progression through a complex regulatory network, not just its ER co-activator activity. To fully understand the role of NCOA7 in breast cancer progression, notably in TNBC progression, additional investigation is required on the functions of NCOA7 other than those required for activation of ER transcription. More importantly, its functions for anti-oxidation and vesicle trafficking in breast cancer cells must be examined.

Therefore, future studies will focus on the role of NCOA7 in regulating ER activation, anti-oxidation and vesicle trafficking processes. This will explore the association of NCOA7-mediated ER regulation, anti-oxidation and/or vesicle trafficking signaling pathways with breast tumor growth and metastasis. By performing these studies, it is expected that the molecular mechanism underlying the promoting effect of NCOA7 on breast cancer progression will be revealed. In addition, NCOA7 will be established as a poor prognostic biomarker and a target molecule for breast cancer therapy.

The nuclear receptor co-activator protein NCOA7 is a potential prognostic biomarker of breast cancer, a possible driver protein for breast cancer progression and a potential target for anti-metastatic therapy for advanced breast cancer.

## Supplementary Material

Supporting Data

## Figures and Tables

**Table I.** Association of NCOA7 expression with clinicopathological parameters in total breast cancer tumor samples.

		NCOA7 status, n (%)		
			
Pathological parameter	Total	Positive	Negative	P-value
Age, years				0.914
≤50	91	40 (44)	51 (56)	
>50	150	67 (45)	83 (55)	
Tumor size, cm				0.020
≤3	194	79 (41)	115 (59)	
>3	47	28 (60)	19 (40)	
T stage				0.005
T1	126	45 (36)	81 (64)	
T2/T3	115	62 (54)	53 (46)	
N stage				0.008
N0	155	59 (38)	96 (62)	
N1-3	86	48 (56)	38 (44)	
TNM stage				0.299
I/II	213	92 (43)	121 (57)	
IIIA/B	28	15 (54)	13 (46)	

NCOA7, nuclear receptor coactivator 7.

**Table II.** Association of NCOA7 expression with clinicopathological parameters in triple-negative breast cancer tumor samples.

		NCOA7 status, n (%)		
			
Pathological parameter	Total	Positive	Negative	P-value
Age, years				0.683
≤50	35	17 (49)	18 (51)	
>50	72	38 (53)	34 (47)	
Tumor size, cm				0.060
≤3	73	33 (45)	40 (55)	
>3	34	22 (65)	12 (35)	
T stage				0.104
T1	39	16 (41)	23 (59)	
T2/T3	68	39 (57)	29 (43)	
N stage				0.032
N0	65	28 (43)	37 (57)	
N1-3	42	27 (64)	15 (36)	
TNM stage				0.674
I/II	91	46 (51)	45 (49)	
IIIA/B	16	9 (56)	7 (44)	

NCOA7, nuclear receptor coactivator 7.

**Table III.** Association of NCOA7 expression with clinicopathological parameters in non-triple negative breast cancer tumor samples.

		NCOA7 status, n (%)		
			
Pathological parameter	Total	Positive	Negative	P-value
Age, years				0.648
≤50	56	23 (41)	33 (59)	
>50	78	29 (37)	49 (63)	
Tumor size, cm				0.567
≤3	121	46 (38)	75 (62)	
>3	13	6 (46)	7 (54)	
T stage				0.077
T1	87	29 (33)	58 (67)	
T2/T3	47	23 (49)	24 (51)	
N stage				0.138
N0	90	31 (34)	59 (66)	
N1-3	44	21 (48)	23 (52)	
TNM stage				0.601
I/II	122	46 (38)	76 (62)	
IIIA/B	12	6 (50)	6 (50)	

NCOA7, nuclear receptor coactivator 7.

## Data Availability

The data generated in the present study may be requested from the corresponding author.
