# Supplementary material for: Expression of nuclear receptor co‑activator 7 protein is associated with poor prognosis of breast cancer
Source: Oncol Lett. 2024 Apr 23;27(6):278. doi: 10.3892/ol.2024.14411 (PMC11063752; doi:10.3892/ol.2024.14411)
Supplement: Supporting Data [file Supplementary_Data.pdf]

Table SI. Selected patients and tumor characteristics.

Characteristic	Value
Patients (TNBC/N-TNBC), n	107/134
Median age (range), years	
Total	53.0 (24.0-85.0)
TNBC	53.0 (24.0-85.0)
N-TNBC	53.5 (32.0-85.0)
Tumor size S, cm	
0<S≤3	194 (80.5)
3<S≤6	41 (17.0)
6<S≤10	5 (2.1)
S>10	1 (0.4)
T stage	
T1	126 (52.3)
T2	105 (43.6)
T3	10 (4.1)
N stage	
N0	155 (64.3)
N1	62 (25.7)
N2	12 (5.0)
N3	12 (5.0)
TNM stage	
I	95 (39.4)
II	118 (49)
IIIA	16 (6.6)
IIIB	12 (5)

Values are expressed as n (%) unless otherwise specified. N-TNBC, non-triple-negative breast cancer.

Table SII. Comparison of NCOA7 expression in TNBC with that in N-TNBC samples in each pathological parameter.

Pathological parameter	Total	NCOA7 status, n (%)		P-value
		Positive	Negative	
Age ≤50 years				0.256
TNBC	35	17 (49)	18 (51)	
N-TNBC	56	23 (41)	33 (59)	
Age >50 years				0.023
TNBC	72	38 (53)	34 (47)	
N-TNBC	78	29 (37)	49 (63)	
Tumorsize ≤3 cm				0.315
TNBC	73	33 (45)	40 (55)	
N-TNBC	121	46 (38)	75 (62)	
Tumor size >3 cm				0.007
TNBC	34	22 (65)	12 (35)	
N-TNBC	13	6 (46)	7 (54)	
T1				0.241
TNBC	39	16 (41)	23 (59)	
N-TNBC	87	29 (33)	58 (67)	
T2/T3				0.257
TNBC	68	39 (57)	29 (43)	
N-TNBC	47	23 (49)	24 (51)	
N0				0.191
TNBC	65	28 (43)	37 (57)	
N-TNBC	90	31 (34)	59 (66)	
N1-3				0.023
TNBC	42	27 (64)	15 (36)	
N-TNBC	44	21 (48)	23 (52)	
TNM I				0.370
TNBC	27	10 (37)	17 (63)	
N-TNBC	68	21 (31)	47 (69)	
TNM II				0.157
TNBC	64	36 (56)	28 (44)	
N-TNBC	54	25 (46)	29 (54)	
TNM IIIA/B				0.395
TNBC	16	9 (56)	7 (44)	
N-TNBC	12	6 (50)	6 (50)	

NCOA7, nuclear receptor coactivator 7; N-TNBC, non-triple-negative breast cancer.
